# Full Spectral Range Raman Signatures Related to Changes in Enameling Technologies from the 18th to the 20th Century: Guidelines, Effectiveness and Limitations of the Raman Analysis

**DOI:** 10.3390/ma15093158

**Published:** 2022-04-27

**Authors:** Philippe Colomban

**Affiliations:** MONARIS UMR8233, Sorbonne Université, CNRS, Campus P. et M. Curie, 4 Place Jussieu, 75005 Paris, France; philippe.colomban@sorbonne-universite.fr or philippe.colomban@upmc.fr

**Keywords:** Raman microspectroscopy, enamel, porcelain, glaze, metal, authentication, zircon, fluorescence, luminescence, rare earth

## Abstract

This study investigates the comparison of the Raman signatures of different phases used in underglaze, inglaze and overglaze decors of selected European, Chinese and Japanese porcelains and enameled metalworks, which are particularly representative of technological developments in enameling. Specifically, the article deals with the main structural types or host networks (corundum/hematite, spinels, zircon, cassiterite, pyrochlore, apatite, sphene, etc.) used for colored enamels on porcelain, earthenware or metal rather than considering all types of pigments and opacifiers. According to the results, Raman microspectroscopy allows identifying of the fingerprint spectra of milestone technologies and represents a simple and rapid tool for detecting copies. Particular attention is paid to the information deduced from the examination of the associated ‘background’ and signatures from electronic transitions induced by uncontrolled traces or voluntary addition of rare earths (luminescence). The relationship between the grinding procedure and Raman signature is also discussed.

## 1. Introduction

The identification of characteristic phases used in the raw materials of ceramic and metalworking can be used as a milestone, regarding a specific period or recipe. Contrary to works of oil painting where the restoration can utilize “modern” pigments, it is very rare for enameled objects to be restored hot by heating. This was done in the 19th century for certain enameled objects on metal (firing is fast and only the surface layers reach the top required temperature) and exceptionally for white porcelains on which a polychrome décor was applied on a body made many years before. Phase identification can thus provide very valuable arguments for authentication and dating of enameled artifacts.

Non-invasive analysis techniques using electromagnetic radiation which interact with electronic levels, differing according to the energy of light, provide varied and rather quantitative information on matter. Raman microspectroscopy, taking advantage of the prodigious developments in laser sources and CCD detectors for the last decades, is widely used in the analysis of materials and objects of cultural heritage [1,2,3,4,5,6,7,8,9,10,11,12] and particularly enameled objects, both in the laboratory and in situ measurements using portable instruments. The Raman intensity is a function of the polarizability of the chemical bonds and the phases whose chemical bonds are strongly covalent displays of intense Raman spectra. Indeed, the stable phases at high temperature based on chemical elements linked by covalent bonds constitute the matter of glass (i.e., glassy silicate) and ceramics. In addition, the colored character of the enamel (a glassy coating powder deposited on a non-porous substrate and then fired) and the glaze (a glassy coating powder deposited on a porous substrate and then fired with the substrate) leads to a cautious choice of the laser wavelength for the resonant Raman phenomenon which improves the detection limit of the coloring phases (chromophores), even in very small amounts. The illumination by a laser not only generates Raman scattering but excites the electronic levels of lower energies, leading to the emission of the fluorescence spectrum, in particular that of electronic transitions of traces of transition metals (e.g., chromium) [13] and rare earths [14]. Furthermore, the colored character of the material analyzed modifies the continuous background due to the selective absorption of scattered light. The presence of nanoparticles with a characteristic plasmon also modifies the spectral signature [15,16]. Consequently, the totality of the spectral signature presents information from a few cm^−1^ to 4000 cm^−1^ and even more (overtones and combination bands enhanced due to resonance Raman effect).

In this study, we address the full Raman spectral signatures of enamels/glazes representative of the 18th century (blue associated with lead arsenate, borax-based blue, arsenate or cassiterite white, Naples yellows, red/rose also called Cassius purple or Perrot ruby), the 19th century (chrome/Victoria green, cobalt aluminate blue, ordered spinel black, sphene pink) and the 20th century (zircon white, zircon blue, cassiterite blue, cassiterite pink) enameling technologies. An extensive review of the variety of pigments used since antiquity has already been published. [17] The most characteristic Raman signatures could be useful as a guide for the detection of anachronistic pigments in copies/fake artifacts [12], especially those produced during the second half of the 19th century and the first half of the 20th century. Indeed, the ‘copies’ made in the 19th and the beginning of the 20th century, were generally made with processes quite close to the oldest ones with care and moreover they entered the collections at the same time with the older pieces. Therefore, their identification is more difficult.

## 2. Materials and Methods

### 2.1. Objects

**Figure 1:**
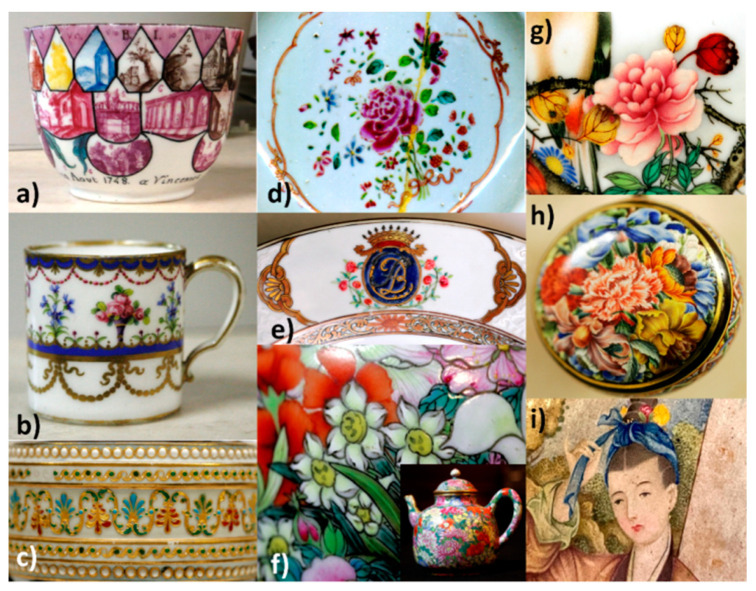
Selection of 18th century objects representative of the variety of colored decorations obtained with enamel on porcelain or metal: (**a**) palette cup (MNC6638, Sèvres), soft-paste porcelain from Vincennes Factory, 1748, adapteded with permission from ref. [21], 2018, Elsevier; (**b**) coffee cup (MNC8102.1, Sèvres), soft-paste porcelain from Sèvres Royal Factory, 1785, adapted with permission from ref. [21], 2018, Elsevier; (**c**) vase decorated by Joseph Coteau, 1783, Comte d’Artois Factory (OA7739, Louvre) [22]; (**d**) *Famille rose* plate, Chinese factory (Private Coll.), circa 1770 [23]; (**e**) ‘*Chine de commande*’ porcelain plate, circa 1740–1750 (R1025, Louvre) [23]; (**f**) ‘Thousand flowers pattern’ tea pot porcelain, Jingdezhen Imperial Factory, 2nd half of 18th century (F1429C, Fontainebleau) [24]; (**g**) Chinese porcelain bottle (TH457, Louvre), Qianlong reign [24]; (**h**) enameled watch, end of 17th century–beginning of 18th century, Swiss factory (OA10079, Louvre), adapted with permission from ref. [15], 2020, Elsevier Masson SAS; (**i**) ewer, with painted (detail) and cloisonné enamel on gold body, 2nd half of 18th century (F1467.1, Fontainebleau), Qianlong Imperial workshop [25,26]. The collections and the inventory number of the objects are indicated. More information is available in the references.

**Figure 2:**
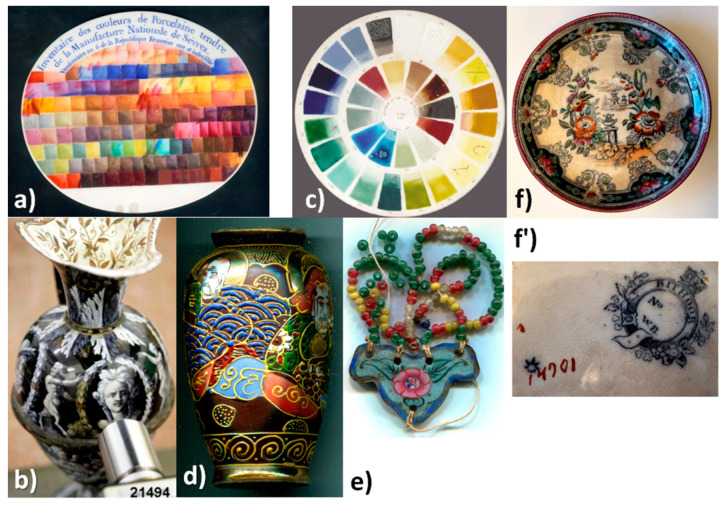
Selection of objects representative of the variety of colored decorations obtained with enamel on porcelain or metal during 19th century: (**a**) color palette (MNC2003, Sèvres), soft-paste porcelain enamels from Sèvres Factory, 1798, reproduced with permission from ref. [2], 2001, J. Wiley & Sons, Inc.; (**b**) painted enameled metal ewer by Jacob Meyer-Heine, Sèvres workshop, middle of 19th century (MNC21494, Sèvres), adapted with permission from ref. [27], 2010, J. Wiley & Sons, Inc.; (**c**) color palette, hard-paste PN porcelain ‘muffle biscuit’ enamel from Sèvres Factory, after 1884, 1050 °C firing temperature reproduced with permission from ref. [2], 2001, J. Wiley & Sons, Inc.; see reference [21] for compositions; (**d**) Satsuma small vase, end of 19th century (Private Coll.); (**e**) enameled metal pendant, Guangdong workshop, 19th century (Private Coll.); (**f**,**f’**) British faience with Chinese-like ‘Indian’ garden pattern, W. Brownfield Factory, Cobridge, UK, circa 1870–1880 (Private Coll.). More information is available in the references.

**Figure 3:**
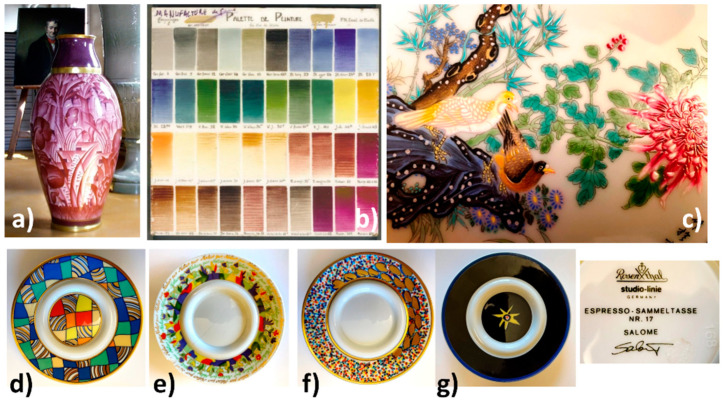
Selection of 20th century objects representative of the variety of colored decorations obtained with enamel on porcelain: (**a**) vase ‘*Inde*’ designed by F. Aubert and J. Beaumont for the *‘La Jungle’*, Exposition Coloniale, Paris, 1931 (MNC2013.1.1, Sèvres), reprinted with permission from ref. [28], 2003, J. Wiley & Sons Inc.; (**b**) color palette for PDN hard-paste porcelain enamel fired at 880 °C from Sèvres Factory, reprinted with permission from ref. [2], 2001, J. Wiley & Sons Inc.; see also [29] for details about compositions; (**c**) Chinese modern copy of Qianlong-style porcelain plate, circa 1995 (Private Coll.); (**d**–**g**) coffee cup saucers from Rosenthal studio-line (Germany): (**d**) Cupola, S. Kreuzer No. 30; (**e**) Somi Jahrestasse 1999; (**f**) Salome No. 17; (**g**) O. Alt No. 7 (Private Coll.). More information is available in the references.

Figure 1, Figure 2 and Figure 3 present the characteristic ceramics and enameled metalworks used as a reference of representative spectral signatures. The selection was made on the basis of hundreds of objects and shards analyzed in the laboratory and in situ measurements for the last twenty years [1,2,3,4,5,15,16,17,18,19,20,21,22,23,24,25,26,27,28]. Artefacts have been selected with the following criteria: variety of colors, structural types of pigments, substrates, firing temperature, etc. In previous studies the attention was focused on the fundamental modes. Some of them have already been analyzed in the standard spectral range and the reader can refer to previous publications mentioned in Table 1. In these previous works, attention was paid to the vibrational signature (internal and external modes) characteristic of the phases without paying attention to the associated ‘background’ up to 4000 cm^−1^. The full spectral range is considered here.

### 2.2. Raman Microspectroscopy

Artefacts were analyzed in the laboratory using a Labram HR800 spectrometer (HORIBA Scientific Jobin-Yvon, Longjumeau, France) excited by an Ar^+^ ion plasma laser Innova I90C 6UV (Coherent Inc., Santa Clara, CA, USA) allowing multiple wavelengths from UV to red. The 457.9 nm line was used with approximately 0.1 to 0.3 (colored enamels) to 2 mW (glaze and light-colored enamels), and exceptionally 5 mW (for the porcelain paste) power of illumination at the sample surface analyzed using long working distance (LWD) 50× or 100× microscope objectives (Olympus Corp., Tokyo, Japan). The 600 g/mm grating was used in order to record a spectrum with a large spectral window (confocal hole: 200 µm). Analyzed spots are about 5 × 5 and 2 × 2 µm^2^, respectively; the in-depth penetration is similar for the colorless and poorly colored glazes, less for dark colored areas. It is well established that blue (or violet) laser excitation is the most efficient to record the Raman fingerprint of silicates [18,19,30,31,32,33]. However, 514.7 nm was also used but a higher power of illumination (up to 10 mW at the sample) is required to obtain ‘nice’ spectra of a porcelain body. Indeed, many instruments are excited with a YAG 532 nm laser and control of the Raman spectra recorded under the green laser with the same Raman set-up is very useful [7,10]. Unless otherwise mentioned, spectra shown in the paper have all been recorded using the blue line. The recording times typically vary between 1 and 100 s with 100-3 accumulations to improve the signal to noise ratio.

## 3. Effectiveness, Limitations and Practical Utility of Raman Analysis

Raman analysis is often defined as a molecular or structural analysis because its spectrum consists of a set of peaks corresponding to the fundamental, internal (stretching and deformation modes) and external (translations, librations and lattice modes or Boson peak) modes of vibration in the vibrational ‘units’ [14,30,31,32,33,34,35,36,37,38]. These vibrational units are the ‘molecular’ entities (i.e., linked by strongly covalent bonds: Si-O, P-O, B-O, etc.) and the ‘isolated’ ions (i.e., strongly ionic) constituting the structural unit analogous to the crystal unit-cell determining the X-ray diffraction pattern. The internal modes can be considered as a fingerprint of the chemical bonds constituting the material whereas the external modes characterize its structure. For a non-absorbent material (i.e., a material that does not absorb the exciting laser wavelength), the standard Raman effect gives a spectrum where only the fundamental modes are visible. On the contrary, Raman analysis of a colored material with laser excitation corresponding to the absorbed color (i.e., complementary to the visible one) will lead to a resonance Raman spectrum where the harmonic modes and combinations are enhanced and become visible [14,28,38]. The number of Raman peaks (as well as their polarization, i.e., the orientation set-up of crystal/grain versus the electric vector of the laser beam) is determined by structural symmetry (site and lattice symmetry) [14,28]. Under the resonance condition, only the modes related to the chromophores are enhanced and the information is thus limited in comparison with that obtained by a Raman spectrum recorded under the non-resonance condition. Furthermore, the relative intensity of the bands and exact wavenumber depend on the wavelength of laser excitation [28].

Enamels are layers of amorphous silicate material (a glass), ‘pure’ (i.e., transparent) or mixed with chromophores [2,16,17,18,29,30,31,39,40]. The latter may include: (i) ions comprising electronic transitions in the visible range, i.e., with incompletely fulfilled 3d (transition metals) and 4f (rare earths) electronic layers; (ii) metallic (Au°, Cu° or Ag°) [16] and semiconductor (CdS, CdSe) [35,36,37,38,39,41,42] nanoparticles absorbing in the visible spectral range; and (iii) crystalline phases colored by these ions or nanoparticles. These coloring species can be formed during the firing process by precipitation or dispersed in the precursor before firing. Their high chemical and thermal stability allow them to not be destroyed/modified by the molten glass during the firing of the enamel [18,19]. Due to the variable efficiency of coloration, the wt% of the coloring agent varies between less than 0.5 wt% (e.g., Co^2+^ ions, metal nanoparticles) and more than 5 wt% (Fe_2_O_3_, Naples yellow, etc.).

As explained in the literature [2,30,31,32,33,34,35,36,37,38], the measured Raman spectrum will be the superposition of the contributions of amorphous (silicate matrix) and crystalline phases (precipitates, pigments, relics from the raw materials) present in the volume of illuminated material (a few to several hundred µm^3^). However, since the intensity of each Raman signature is extremely different according to the phases present in the volume of material in which the laser beam is focused (mainly a function of the covalence of the bonds, the number of electrons involved in the bonds and the laser wavelength) [14], the spectrum will only inform about the presence of some of these phases. For instance, tridymite (a variety of silica) is hardly detected by Raman microspectroscopy but well identified by X-ray diffraction. Certain phases do not have a Raman spectrum observable under standard analysis conditions (e.g., metallic nanoparticles) and they can be detected with the generation of other optical phenomena, such as modification of the relative peak intensity, wavenumber shift of the matrix signature [16,28], continuous background of broad fluorescence for metallic nanoparticles characterized by a plasmon [15,16] and series of narrow fluorescence peaks for rare earth and some transition metal ions [13,43].

Raman excitation with a blue laser is particularly well suited for the study of oxides, in particular amorphous silicates, but mobile instruments using this excitation are necessarily less compact than those excited by green and a fortiori by red or infrared [7,8,9,10] lasers. Furthermore, on-site analyses do not have the performance level of those carried out in the laboratory.

## 4. Technological Evolution of Enameling: A Brief Overview

The preparation of enamels has greatly evolved since the 15th century (Renaissance) with the development of European artisanal productions including the empirical knowledge of chemical processes and their circulation by means of the recipe books and later with the huge advances in industries and chemical technologies during the 19th century [17]. Globalization of the enameling technology starts during the 17th century with the long-distance trips and trade of European experts [12,21,22,23,24,25,26].

From the very beginning of glass production, the coloring of glasses was mainly done using transition ions dissolved in the glassy silicates (so-called ‘transparent colors’) [2,18,21,30,31]. In that case, the Raman signature results from the glassy silicate hosting the coloring ions which do not have specific vibrational contribution. However, since antiquity, certain colors have been obtained with crystalline phases formed by precipitation during the firing of glass as a result of the saturation in certain elements (e.g., Sn giving precipitation of cassiterite (SnO_2_), Pb and Sn giving precipitation of lead-tin yellow (Pb_2_Sn_2_O_6_, also called Naples yellow)). Alternatively, a specific preparation method includes mixing ‘*anima*’ (rich in coloring agent) with ‘*corpo*’ (the glassy matrix).

The demand for new colors to be able to create complex decorations by mixing ‘colors’ for thin colored layers (a few tens of microns) has led to specific syntheses and taken advantage of the possibilities of purifying raw materials, mainly since the 18th century [18]. It was only in the second half of the 19th century that ‘chemicals’, i.e., oxides, nitrates, sulfates and carbonates, began to replace ‘natural’ or poorly processed raw materials (Thiviers sandstone, alquifoux, Armenian bole, etc.) [4,18,19,20,44,45] as well as residues from other production processes (‘*battitures*’ (scales forgings), ‘*smalt*’ or ‘*saffre*’ (modified slag of silver/bismuth production), etc.) [2,29,46,47] for the preparation of coloring enamels while the natural raw materials remained in use for the silicate matrix (quartz, flint, feldspar, pegmatite, etc.) [19,20,29,39,40,46,47]. Regarding the use of natural ‘stone’, the procedure of crushing and grinding was a difficult and energy-consuming step, partially overcome by the use of powders prepared by thermal treatment (ashes, heated flint, etc.) [20]. The use of chemicals, generally produced in the form of fine powder, becames common in the (first half of the) 20th century and then the enamels were no longer prepared by artisans and factories but sourced as ‘ready to use’ from international companies specializing in the production of enamels [39,40]. Mixtures of raw materials with warranted characteristics specially designed for use in the preparation of ceramics then became available which eventually led to mass production. Here, it is worthy to note that selected natural raw materials such as Thiviers sandstone, Armenian bole, etc., still offer ‘*natural*’ nanosized coloring agents [20]. For instance, Thiviers sandstone also called ‘*Grès de Thiviers*’ is composed of alpha-quartz grains cemented by goethite (alpha-FeOOH), leading to red hematite during the firing process [45]. Meissen craftsmen were the first to observe that overground hematite produced a nice red-to-orange pigment, due to the shift of the optical gap induced by the nanometric size of the hematite grains [5,20].

**Figure 4:**
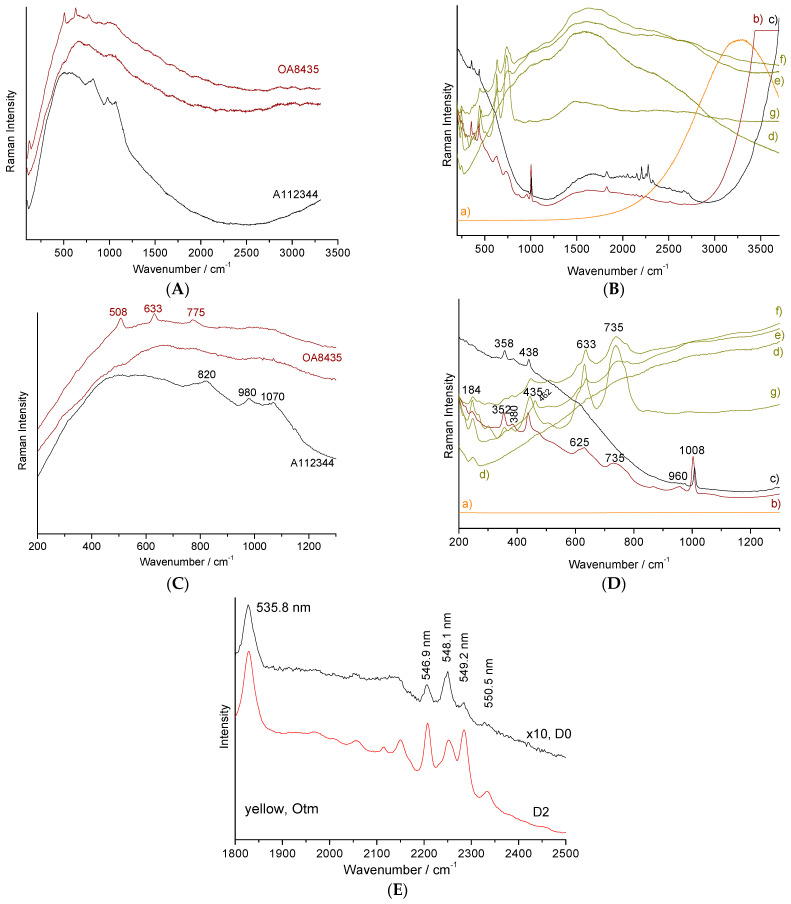
Representative spectra having a strong fluorescence contribution: (**A**), 532 nm laser excitation; red enamel on metal (two spectra, OA8435 18th century watch [15], Louvre Coll.; zoom in (**C**) and on glass (A112344 figure, Orléans City Museum Coll. [18]) obtained with a dispersion of Au° nanoparticles; (**B**) 457.8 nm excitation (zoom in (**D**)): (a), red overglaze on porcelain colored by CdS_1-x_Se_x_; (b,c) pale yellow glaze on porcelain (Figure 3e, Somi cup); (d–g) yellow glaze on porcelain colored by addition of praseodymium and opacified by addition of cassiterite and yttrium-stabilized zirconia. A zoom of (b) and (c) pale yellow glaze in the 1800–2500 cm^−1^ range is given in (**E**) (power at the sample, 5 (D0) and 0.05 (D2) mW).

**Figure 5:**
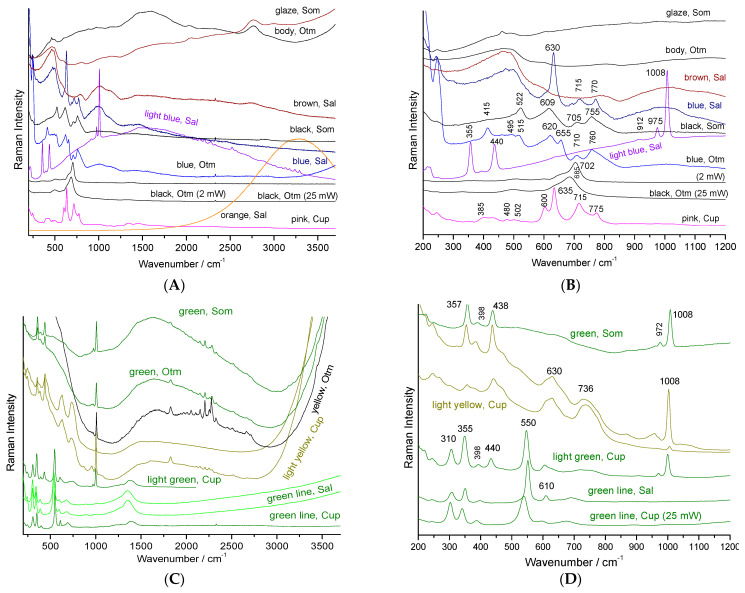
Representative Raman spectra and background recorded on 20th century porcelain with complex colored décor (Figure 3d–g): (**A**) and (**C**): full spectral window; (**B**) and (**D**): zoom in the 200–1200 cm^−1^ range. Som: Figure 3e; Sal: Figure 3f; Cup: Figure 3d; Otm: Figure 3g. Full spectral range is given on the left and a zoom between 200 and 1200 cm^−1^ on the right side.

**Figure 6:**
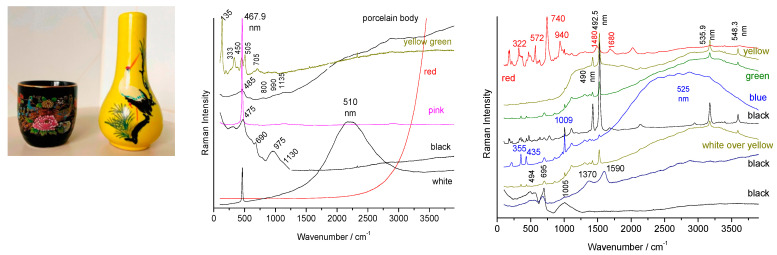
Representative Raman spectra and background recorded for two porcelains presented on the left, respectively made in Japan and Vietnam in the 1980s (see text).

**Figure 7:**
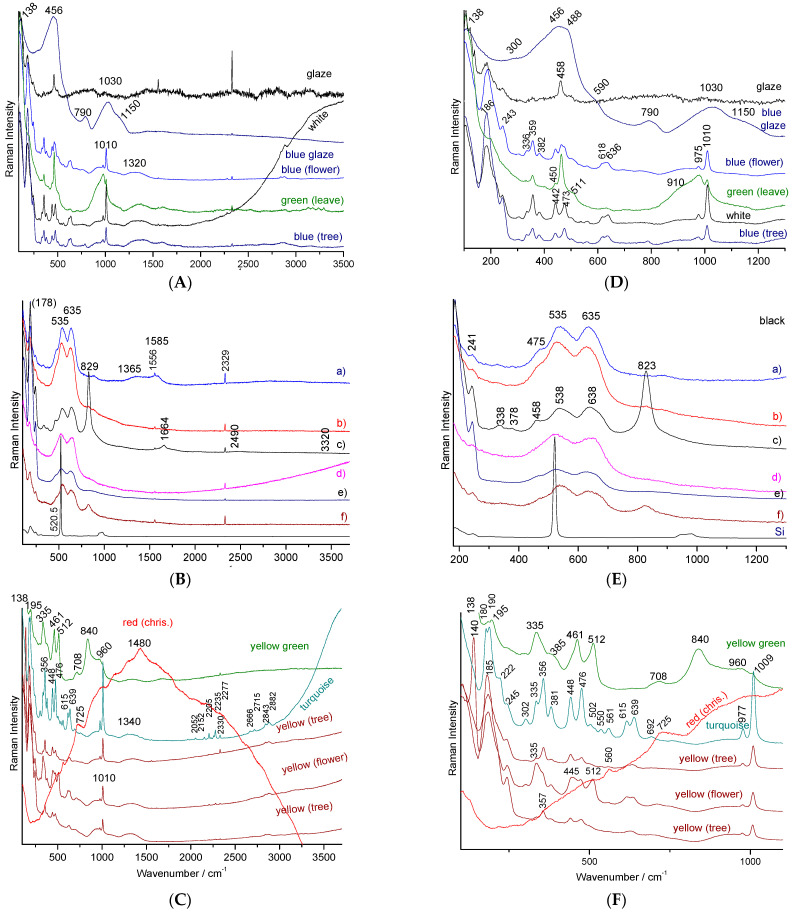
Representative Raman spectra recorded on a 20th porcelain with a design copying Qianlong-style production (Figure 3c); full spectral window is given on the left (**A**–**C**) and a zoom on the spectral range of the fundamental mode is given on the right (**D**–**F**): (**A**,**D**) colorless glaze, blue glaze, blue, green and white overglaze; (**B**,**E**) black lines and areas; (**C**,**F**) turquoise, yellow and yellow-green overglaze (See text for explanation).

**Figure 8:**
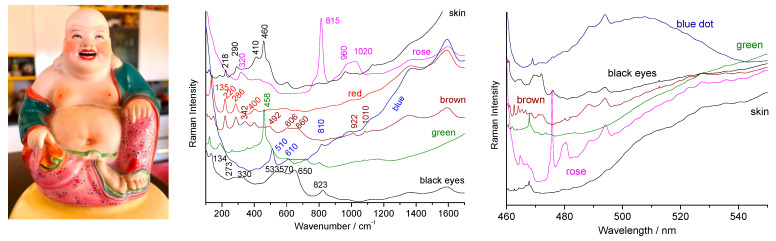
Representative Raman spectra recorded on a Chinese porcelain Buddha figure from the 1960s; right full range spectra plotted in absolute scale (nm); a zoom of the spectra is given on the left (wavenumber scale); colored area examined are given.

**Figure 9:**
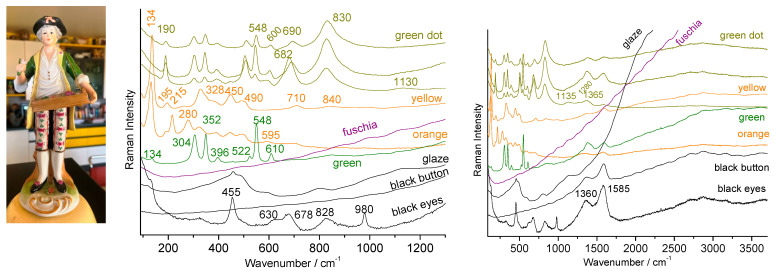
Representative Raman spectra recorded on porcelain figure from the end of the 19th century or beginning of the 20th century (unknown German factory); a zoom of the spectra below 1300 cm^−1^ is given on the left; colored areas are given.

**Figure 10:**
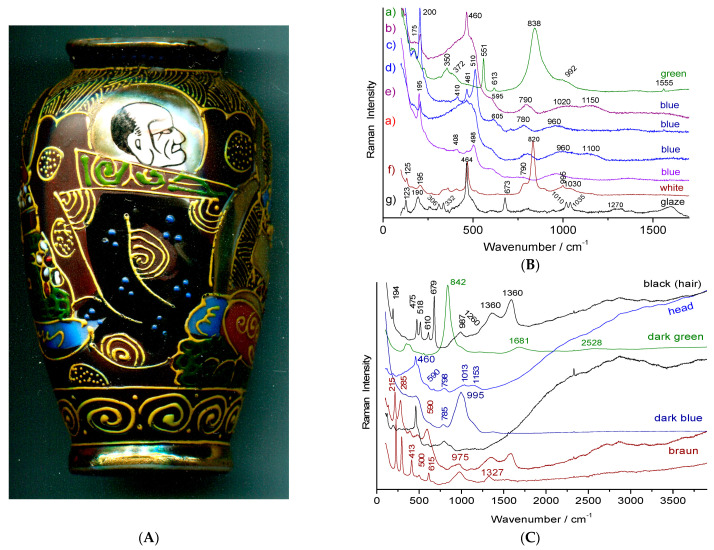
Representative Raman spectra recorded on Satsuma enamel vase from the end of 19th century (**A**) and Figure 2d; (**B**) 514.5 nm laser excitation; (**C**) 457 nm laser excitation; colored areas examined are given.

There are several significant written sources which give a detailed view of the preparation of enamels/glazes based on recipes in use during the 19th century. The most complete source from our point of view involves the book of Théodore Deck [46] which displays the formulae used by this great ceramist to make ‘replicas’ of ancient masterpieces representative of the world productions. In one of the volumes of the famous Roret Technical Encyclopedia series, H. Bertrand [47] described the formulae of enamels on glass, porcelain as well as on metal while Antoine d’Albis [29], the former technical manager of French National Factory of Sèvres, published a compilation of the formulae for preparing enamels and glazes used at the Sèvres factory over several centuries. On the other hand, the book by Richard A. Eppler and Douglas R. Eppler published in 2000 [39] presents a complete survey of contemporary enameling technologies. Based on these works and our previous publications [2,4,5,15,16,17,18,19,20,21,22,23,24,25,26,27,28,41,42,43], Table 2 lists the main structural types of pigments from different historical periods. The different phases are classified according to the criteria used in solid state chemistry and ceramics textbooks [48] for structural types. Indeed, the consideration of symmetry and nature of the chemical bonds indicates that the Raman spectra are very similar for the same structural type. In this case, several mineralogical forms can exist, characterizable by X-ray diffraction, such as in the example of olivines and spinels. Since the differences of the Raman signatures between these mineralogical forms are weak and the observable spectra are strongly influenced by the effects of orientation, it is not possible, nor reasonable, to specify each possible form. Similarly, the formulas indicated have been simplified and only the spectral range corresponding to the most characteristic Raman peaks is indicated. Representative Raman signatures of these pigments are presented in Figure 4, Figure 5, Figure 6, Figure 7, Figure 8, Figure 9 and Figure 10. Table 2 gives an illustration of the large number of new phases with complex composition developed during the 19th and especially the 20th century. It should be noted that complex natural phases, such as lazurite ((Na,Ca)_8_(AlSiO_4_)_6_(SO_4_,S,Cl,OH)_2_) and its neighboring phases [49,50], hematite having Fe ions substituted by Al or Ti ions [51], spinels based on Fe, Cr and Mn [29,51,52,53] were also used, undoubtedly after visual selection of the stone/mineral, well before the 19th century. For instance, the potters and glassmakers of the Lagid Dynasty (1st century BCE), then the Iranians, Normandians and Mamluks (13th–14th century, blue enamels) [50] used lapis lazuli as a blue pigment, sometimes mixed with cobalt. During the 18th century, even Johann Friedrich Böttger at Meissen [5] revived its use. Additionally, Iznik potters (from the 15th century) used complex natural spinels and chromites for the realization of the black lines of polychrome decorations [4].

The phases used as pigments should not only be refractory (melting temperature > 1200 °C, at least ~100 °C above the firing temperature) but also made of compact packing structures to guarantee the low chemical reactivity required for their stability when the enamel is brought to the molten state to perfectly coat the substrate [18,19,39]. The high compactness also gives them a rather low coefficient of thermal expansion, which allows their perfect inclusion in the amorphous silicate matrix. Controlling the difference between the thermal coefficients of expansion-shrinkage of the enamel layer and the support is crucial to avoid defects such as cracking or peeling [19,39]. The enamel layer should be slightly under compressive stress after firing. However, metals—and compositions containing quartz—exhibit an expansion approximately double that of porcelain rich in mullite [5,18,19]. The composition of the silicate matrix in which the coloring agents are dispersed must therefore be adapted both to the firing temperature and the nature of the substrate. The firing temperature therefore ranges between ~650 and 700 °C (enamels on metal, last overglaze deposited on already glazed/enameled surfaces) and ~1400 °C (glaze fired with porcelain body) [19,39,43,44,45,46,47,48]. For example, in Figure 2 and Figure 3 the color palettes for muffle firing at 1050 °C and 880 °C are presented, respectively; the range of these later temperatures being used mostly for the firing of enameled porcelain. In fact, the color palette is reduced with the increase in the firing temperature, because many pigments are not stable enough. Note that for the manufacture of the most complex decorations shown in Figure 1, Figure 2 and Figure 3, several firings are necessary [18,19,44].

## 5. Results and Discussion

We will here consider complete spectral signatures (i.e., not only fundamental vibrational modes measured below ~1500 cm^−1^ but also the ‘background’ up to ~3700 cm^−1^) obtained from objects going back in time, starting from the analysis of contemporary objects (Figure 3). As all the spectra contain information, especially for ‘modern’ enamels, the spectra are given ‘as recorded’, without baseline subtraction [54]. To identify the spectral signature of the main coloring agents, the representative spectra of the porcelain substrate and a colorless glaze are shown in Figure 4 and Figure 5.

The spectrum of a glassy silicate consists of two broad bands around 500 and 1000 cm^−1^ corresponding to the modes of deformation (bending) and elongation (stretching) of the Si-O bond of the SiO_4_ tetrahedron forming the amorphous silicate network [31,32,33,34,35,36,37,38,54]. The spectrum of mullite, the major crystalline phase of porcelain body (3Al_2_O_3_·2SiO_2_) is very weak and difficult to record [1,55]. The spectrum of associate glassy phase is also weak (Figure 5).

Amorphous silicates have mainly been studied by geologists and the compositions of natural glasses are in fact quite limited (obsidians very rich in silica, calcium glasses, etc.) [35]. It is the same for the compositions studied by solid physicists (pure silica and borosilicates) [36]. Vibrational analyses have mainly focused on the understanding of the origin of the different components of the SiO_4_ stretching band. For instance, Mysen et al. [34] associated each component with the different types of SiO_4_ tetrahedron forming the amorphous network. The study of the wide variety of compositions used to enamel the different substrates led us to highlight that the main spectral information, such as the Raman intensity [33], neglected by the previous authors, made it possible to evaluate the polymerization degree of the SiO_4_ tetrahedron, a parameter directly related to composition and melting temperature [31,32,33].

Added to the signature of glassy silicates, are generally small contributions of un-reacted grains of quartz (main narrow peak at ~464 cm^−1^, usually downshifted to ~458–460 cm^−1^ due to the stress of the glaze matrix), feldspar (narrow peak at ~510 cm^−1^) and more rarely cristobalite, tridymite, feldspar/plagioclases, rutile, anatase, wollastonites (either alpha, beta or both) and calcium phosphate [1,2,3,4,5,21,22,23,24,25,26,27,28,29,30]. For the paste, the spectrum contains the weak signature of mullite (3Al_2_O_3_·2SiO_2_), which differs little from that of an amorphous silicate but with some characteristic peaks above 600 cm^−1^ [1,2,5,55], and those of the phases present, such as those in the glaze, plus rarely brookite and zircon. However, a clear difference exists concerning the spectral signature beyond 1300–1500 cm^−1^. While the spectrum is rather flat or shows fluorescence steadily increasing towards high wavenumbers in objects from the 19th century and earlier, signatures of recent objects (>1960s) generally show additional peaks of fairly well resolved fluorescence (they shift when the spectra obtained under blue and green laser excitation are compared). Actually, rare earths are also used as coloring agents [39]. It is likely that the use of electrofused zirconia beads as a grinding agent in replacement of natural flint pebbles or steel balls—the purity of ‘zirconia’ beads is poor and the material contains many rare earth impurities—has resulted in rare earth contamination, which induces quite well resolved fluorescence peaks. The choice of grinding mode by attrition is motivated by its higher efficiency and the fact that the low zirconium pollution has no deleterious effect on the color. Less than 0.1 wt% of rare earth content is sufficient to give a clear signature.

Rare earth ions having unfilled f-orbitals exhibit luminescence with f-f and f-d electronic transitions when excited with appropriate energies [43,56,57,58,59]. When this occurs, a specific signal is measured by the spectrometer provided that the grating allows for collection of the light at the emitted wavelength and the integration time is long enough when pulsed laser source is used. The fluorescence signal obtained received the attentions of mineralogists [56,57] and engineers [13,58] because the signal informs about the local site symmetry and luminescent pigments have many applications (paints, screens). What is seen as a problem with laser-induced fluorescence in Raman spectroscopy in many cases, here it can be used as a tool to discriminate colored coatings prepared by different routes. Typical rare earth ions producing series of rather ‘narrow’ peaks in the 300–2000 nm range are Eu^3+^, Nd^3+^, Tb^3+^, Ho^3+^ and Sm^3+^ [59]. 

The first use of such information for archeometric purposes was made in the study of Medici porcelain [43,60] as well as selected ancient Japanese, Chinese and German porcelains more recently [61]. The width of the bands, and to a lesser extent their positions, are determined by the structure (symmetry, disorder) of the site. The bands are broad and strongly mixed when the 4f or 3d ions are in an amorphous lattice and offer series of thinner peaks if the host is a crystalline phase [43,59,61]. The spectrum is then strongly dependent on the temperature and firing cycle [61]. The identification of the ions at the origin of the luminescence cannot always be carried out since it is not an easy task. Formation of defects complicates the identification of all transitions, even in a single crystal [59]. Detailed study is beyond the scope of the present work.

### 5.1. Fluorescence Contribution in the 20th Century Pigments

Two types of coloring agents also give strong but very broad fluorescence features: gold nanoparticles (maximum at ~500 nm, i.e., ~500 cm^−1^ for blue excitation) and CdS (~534.5 nm), CdSe (beyond 590 nm) and the solid solution CdS_1−x_Se_x_. Figure 4 shows some representative examples. Indeed, with blue or green laser excitation, the enamels colored by gold nanoparticles (also called Cassius’ purple and Perrot’ ruby) present a strong fluorescence background as shown in the example of the red regions of complex decorations of 18th century watches representing a famous oil panel [15]. Over the spectrum, it is often possible to detect the tiny contribution of the silicate matrix in which the gold nanoparticles are dispersed (typically < 2 wt% gold according to the elemental analyses [15,23]) and even the presence of cassiterite [2] or lead arsenate [15,24,25,26], according to the two different preparation methods of gold nanoparticles [15,16,62]. For instance, cassiterite (SnO_2_) was detected with its very characteristic 635–775 cm^−1^ doublet in some spots of the red enamel on metal (Figure 4, OA8435 artefact, enameled watch [15]) although the broad band at ~820 cm^−1^ (Figure 4, A112344 artefact, soft-paste porcelain [62]) is characteristic of lead arsenate. The broad component of the glassy matrix can be observed at about 1000 cm^−1^. These preparations of colloidal gold by the addition of tin or arsenic are usually considered to have been invented by Johann Kunckel (Germany) and Bernard Perrot (born Bernardo Perrotti; he worked in France, Orléans, but was born in Altare, Italy) [16,62], respectively in the 17th century. However, more precise studies trace these methods to Italian arcanists of the 16th century, in particular Cassius and Libavius and even much earlier to the glassmakers of the Late Roman Empire [16]. 

The spectrum obtained from the yellow glaze colored with praseodymium shows a very wide fluorescence background centered on 1600 cm^−1^ (530 nm) under blue excitation. Depending on the point of analysis, the contribution of the Raman signatures of the opacifiers (Figure 4), cassiterite or stabilized tetragonal zirconia ‘pure’ or doped with praseodymium were detected in variable proportions. However, the matrix most used to host praseodymium ions is zircon (ZrSiO_4_, Table 2). The luminescence signature exhibits a series of clusters of thin peaks (Figure 4E) typically spaced ~2000 or 3000 cm^−1^ apart, so that excitations with different lasers make the clusters of different transitions visible. As shown in Figure 4, the spectrum of a porcelain glaze (Figure 3e) colored in yellow with praseodymium zircon, exhibits a group of fine lines between 1700 and 2500 cm^−1^ under blue excitation. The contribution of other rare earths associated with praseodymium cannot be excluded. It is important to note that using a high laser power makes it more difficult to obtain ‘good’ luminescence spectra. 

The spectrum of zircon is characterized by its symmetric narrow strong Si-O elongation mode at 1009 cm^−1^, its deformation weaker modes at 458 and 357 cm^−1^ (see further) and it is likely that the narrow luminescence peak series appearing at 546–550 nm arise from rare earth ions trapped in the zircon lattice. Except for the two works cited above [43,60,61], to our knowledge, no attention has been paid to the use of narrow luminescence peak signature and fluorescence background to classify and authenticate old enameled productions, whatever the substrate used. It is certain that this type of approach can provide new classification tools, in particular using multivariate analysis. Broad bands at ~430, 620 and 720 cm^−1^ (Figure 4, left side, spectra e–g) could be assigned to a titanium-based phase with pseudo-brookite or rutile structure.

### 5.2. 20th Century Pigments

The plate which a part of the decoration is presented in Figure 3c, attempts to use the color palette of productions characteristic of the reign of Qianlong (Qing Dynasty). If the pattern roughly mimics this type of 18th century decor well, the simple visual examination of the overglazes, much thicker than in the original productions, classifies this plate as ‘in the manner of’ and not as a fake. However, it is interesting to identify the technical solutions used. The glaze spectrum is difficult to obtain and, in most spectra only the quartz signature is observed (458 cm^−1^, Figure 5A). The very narrow peaks at 1555 and 2330 cm^−1^ are due to gaseous molecules of O_2_ and N_2_ trapped in bubbles in the glaze. 

Due to the large absorption of the fluorescence emitted by matter colored in blue, very nice spectra almost free of significant background are recorded as usual when the glaze is colored blue [15]. The broad and rather strong band of the Si-O deformation mode is observed around 455 cm^−1^ and the broad weak components of the Si-O elongation are found at ~1030 and 1150 cm^−1^, as usual for a porcelain glaze fired at high temperature. The spectra of the white and blue overglazes also show the thin peaks of zircon. The signature of the overglaze shows an elongation band centered at ~970 cm^−1^, much more intense than the band of the deformation mode, which is characteristic of a glass that is more depolymerized and fired at a much lower temperature than the colorless glaze fired with the body [30,31,32,33]. The presence of weak and narrow peaks between 2000 and 2500 cm^−1^ and 3000 and 3500 cm^−1^ should be noted where the latter are attributed to rare earth luminescence. The luminescence signatures are different for the green and blue overglazes, indicating different rare earth contents. The green areas only show the signature of amorphous silicate and quartz, without the signature of other crystalline phases. This finding is in agreement with the coloration by only Cu^2+^ ions, as expected [2].

The observation of the intense signature of zircon for light blue, green and yellow overglazes indicates the use of zircon, pure and modified by the addition of vanadium, respectively, for white and blue, according to the usual recipe developed in the second part of the 20th century (see recipes in Eppler and Eppler [39]). We also find these characteristic signatures observed on the Rosenthal porcelain saucers, such as the variety of zircon-based pigments for blue, white, yellow, yellow-green and turquoise colors, representative of the productions with complex decoration of the end of the 20th century. The Raman spectrum recorded on turquoise is also very characteristic of the 20th century décor with the signature of zircon and the very well-defined luminescence peak series. Cassiterite pigment was used as the pigment for the pink color (Figure 5A,B).

The two objects presented in Figure 6 are particularly representative of the use of modern coloring agents and their signature is generally dominated by the contributions of luminescence and fluorescence. Thus for the small black cup (Japan), ‘pure’ Raman signatures were obtained for (i) the porcelain paste: a weak quartz peak at 456 cm^−1^ and the wide spectrum with the main components at 485, 800, 990 and 1135 cm^−1^ which are characteristics of a porcelain paste based on mullite and vitreous phase [1,2]; (ii) the black lead enamel with the Si-O stretching mode peaking at 975 cm^−1^ and the bands characteristic of a spinel (690 cm^−1^) [51,52,53,61,62,63,64,65,66] and the Fe-S complex (475 cm^−1^) [67]; and (iii) the yellow-green areas (leaves) which show the spectrum of Pb_2_Sn_2_O_6_ (135, 333, 450 and 505 cm^−1^) [23,25,27]. 

For the other colors (creamy white, red and pink) the luminescence, in particular the narrow peak at 467.9 nm, corresponds exactly to the wavenumber of the main quartz peak (456 cm^−1^) [1], but the other Raman components are totally absent. This makes the confusion between this fluorescence peak and the main vibrational peak of quartz very easy. The components at 510 nm and beyond 600 nm correspond to compositions of the solid solution CdS (yellow)–CdSe (red) [41,42], widely used between 1960 and 1980. The spectra obtained on the yellow flask are also particularly typical with the very present signature of zircon (355, 435 and 1009 cm^−1^) [68] for the colors blue (V: ZrSiO_4_), white, yellow, green and even weakly in the red and black and the broad components of fluorescence (yellow, green) and fine luminescence peaks (490, 492.5, 535.9, 548.3 nm and less intense between these last two peaks). The red spectrum is that of chromium-doped malayaite sphene [28] whose resonant character is seen by the presence of harmonic modes and the combination of bands linked to the chromophore peaks at 740 and 940 cm^−1^ [28]. The spectrum of the black traces shows the signatures of a spinel (695 cm^−1^), and often of carbon (doublet at 1370–1590 cm^−1^) and probably also a little of the Fe-S complex.

Examination of the areas colored in black (Figure 7B,E) shows two types of signatures, both resonant. Indeed, small intensity features at wavenumber positions corresponding to those of the fundamental modes (located below ~1000 cm^−1^) multiplied by 2 or 3 are observed. The black color is also obtained with the use of iron-rich spinel exhibiting a strong peak between about 500 and 700 cm^−1^ [51,52,53,63,64,65,66,69]. The doublet at 535–635 cm^−1^ corresponds to a spinel phase rich in manganese as a pigment while the intense peak at about 830 cm^−1^ indicates the addition of chromium. Further study is needed to catalog the different spinels precisely (Table 2). The strong ~840 cm^−1^ peak detected on the yellow-green areas (Figure 7B) indicates the use of Victoria green (chromium-based garnet), a famous green pigment since the middle of the 19th century [2]. The very particular feature obtained from the red chrysanthemum is characteristic of the fluorescence background generated by gold nanoparticles (Figure 3c) [15]. The additional 1480 cm^−1^ peak arises from the contribution of carbon enhanced by the SERS effect.

Figure 8 presents the characteristic spectra of the enamels of a figurine produced in the 1960s in mainland China before the modernization and internationalization of Chinese industry. Indeed, an enameling recipe can be used for centuries without much evolution. The techniques are therefore traditional and are linked to those of (the end of) the 19th century. The contrast with the 21st century copy of a Qianlong décor (made around 2015) discussed above (Figure 7) is obvious. No use of zircon is observed here. The skin was obtained by leaving the porcelain substrate unglazed which leads to a signature of mullite (peaks at 410, 600 and 960 cm^−1^) and quartz (~460 cm^−1^). The pink enamel is opacified with lead-arsenic apatite (peak at 815 cm^−1^) and the pink color should be due to incorporation of gold nanoparticles according to the fluorescence bump at ~530 nm (Figure 8, right). The green color does not present any signature other than that of the glaze in accordance with the coloration by copper ions. 

The black of the eyes is characterized by the spectrum of a pigment rich in iron and manganese, with the formation of spinel [2,21,22,23,24,25,26,52,53,63,64,65,66,69]. The red is made of hematite [2,51] and the brown was obtained by mixing these two colorations. Note the very high sensitivity of the spectrum to the illumination power which causes oxidation and phase changes from a few to 0.1 mW [51,70,71]. The blue dots show a glaze spectrum, with a weak quartz signal, characteristic of coloring by cobalt ions dissolved in the glaze. No luminescence signal was detected. The Raman signatures are rather similar with those of 19th century productions, either regarding the phase identified and the type of background (absence of series of narrow luminescence peaks). This artifact gives an illustration of how ‘traditional’ recipes have longtime continued use.

### 5.3. 19th Century Pigments

**Figure 11:**
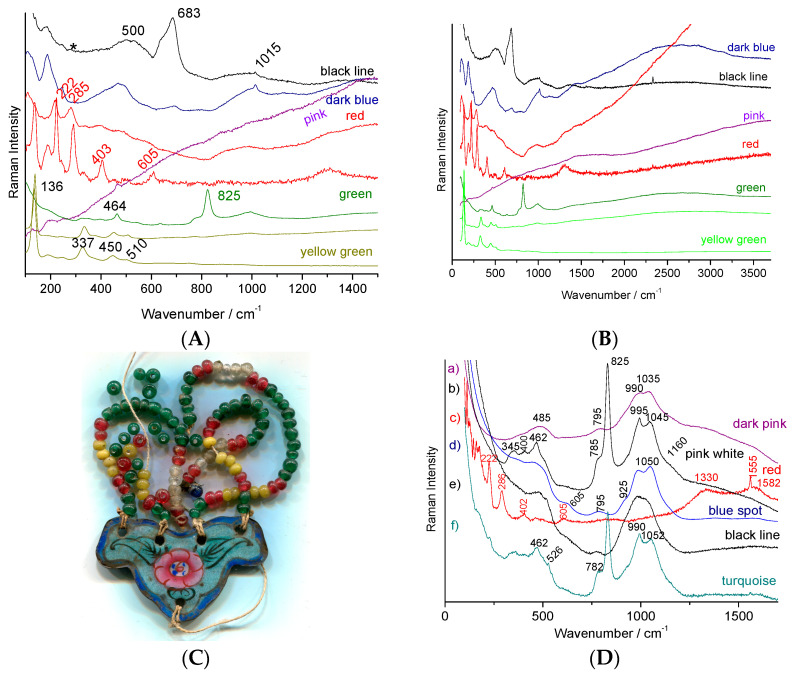
(**A**,**B**) Raman spectra representative of the decoration of a British faience (W. Brownfield factory, see Figure 2f), on the left zoom of the spectral domain characteristic of the fundamental modes; (**C**) photography and (**D**) Raman spectra of a Chinese necklace representative of enamel on copper with enameled pendant (Canton, Figure 2e); colored areas examined are given. Band with a * arise from the filter.

**Figure 12:**
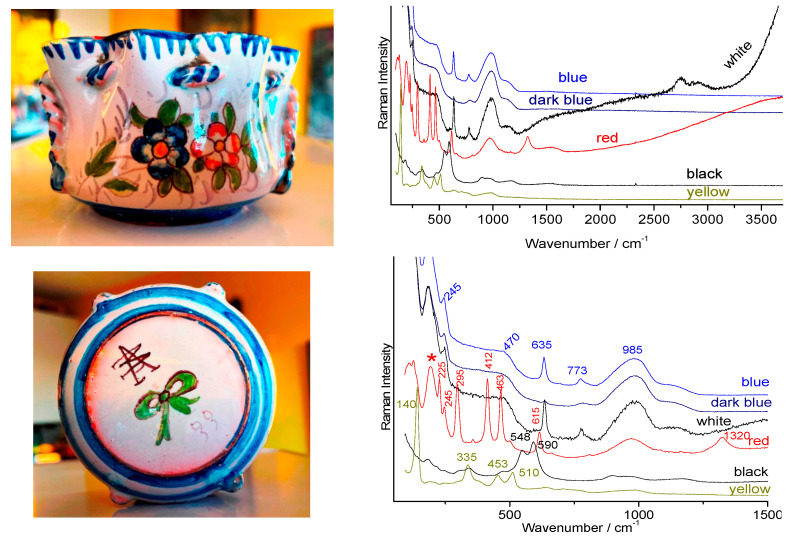
Representative Raman spectra recorded with 514.5 nm excitation on 19th-century Nevers faience (Montagnon factory, Nevers); colored areas examined are given. Band with a * arise from the filter.

**Figure 13:**
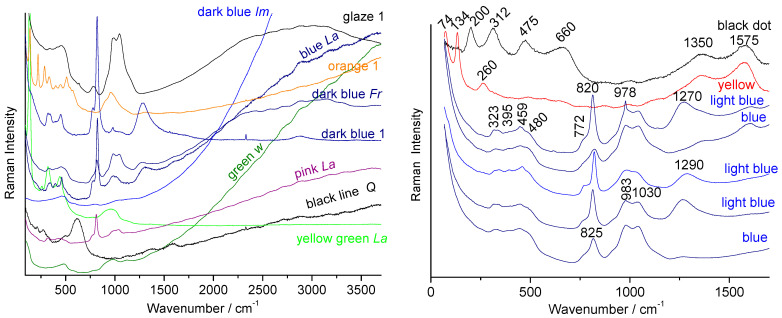
Raman spectra recorded on European (glaze 1, orange 1, dark blue 1), Chinese *Famille rose* (dark blue lm, blue La, dark blue Fr, black line Q, pink La, black line Q and yellow green La) and *wucai* plates (green w) for different colored enamels representative of the 18th century enameling recipes (see reference [23]). Details for *Famille rose* overglaze are given on the right. Note the variation of the Raman spectrum and fluorescence background of blue enamel with color density in the same area.

We will examine the Raman spectra (Figure 9, Figure 10 and Figure 11) recorded on artefacts especially representative of the 19th century enameling technology: a porcelain figure (Figure 9), a vase (Figure 10) made in Satsuma (Japan), a British faience (Figure 11) with Chinese-like ‘Indian’ garden pattern (W. Brownfield Factory, Cobridge), a faience cup (Figure 12) with flower décor made in Nevers (France, Montagnon factory) and an element of an enameled copper necklace in the manner of Canton decorations (China) (Figure 13). The spectra are generally easy to obtain under blue excitation with moderate fluorescence backgrounds and show no thin peaks of rare earth luminescence. Raman spectra show a much greater variety of signatures than those of the 20th century enamel objects. Spectra are like those of Figure 8.

The green color of the porcelain figure (Figure 9) shows the typical spectrum of chromium oxide (Cr_2_O_3_: 304, 352, 548 and 610 cm^−1^ under blue laser excitation; peak position and relative peak intensity slightly change with laser wavelength [2,28]). This pigment only appeared at the end of the 19th century with the mastery of chromium chemistry. For the green points of the bands, in the darker color, we observe the strong chromate band at ~830 cm^−1^, indicating a Victoria green pigment (3CaO·Cr_2_O_3_·3SiO_2_), another typical pigment of the 19th-century [2]. The black was obtained either with carbon or the addition of spinel.

The green color of the Satsuma vase (Figure 10) also shows a typical spectrum of Victoria green (Figure 10B). One can observe, particularly under blue laser excitation, the harmonics (1681 and 2528 cm^−1^, etc.) and combinations of the Cr-O fundamental mode at ~840 cm^−1^. The white lines and dots were obtained with lead(-calcium-potassium) arsenate with apatite structure (narrow ~820 cm^−1^ peak and shoulder at ~780 cm^−1^) [21,22,23,24,25,26], while the black color was achieved with a particularly well-crystallized spinel (683 cm^−1^ for a measurement at very low power of illumination, the wavenumber position decreasing rapidly under strong laser power, Figure 10C). A lead-tin pyrochlore Naples yellow was used for the yellow (intense peak at 138 cm^−1^, not shown) [21,22,23,24,25,26]. The brown color was obtained with hematite. The width of the hematite peaks varies according to the point of analysis which indicates variations in composition (partial substitution of Fe by other ions). This would indicate the use of natural sources for iron oxides and not of chemicals. The spectrum of the dark blue reliefs (lines and points) only shows the signature of a lead glass (intense band of Si-O stretching at 995 cm^−1^, Figure 10B) while that of the light blue areas (Figure 10A) shows that of a glaze with an intense band around 500 cm^−1^ (Si-O bending) and two weak bands at 960 and 1150 cm^−1^ [32,33,34,35]. The main quartz peak (~460 cm^−1^) was detected on certain points. No characteristic pigment peak is (usually) observed, consistent with staining by Co^2+^ ions ‘dissolved’ in the vitreous network. However, at rare points, the signature of spinel CoAl_2_O_4_ was detected (410, 460 and 510 cm^−1^). Similarly for the Victoria green areas, Cr_2_O_3_ peaks are sometimes found (551 and 613 cm^−1^). This indicates the use of pure chemicals and incomplete reaction with glassy coating. 

The comparison of the spectra obtained under green (514.5 nm, Figure 10B) and blue (457 nm, Figure 10C) excitation is interesting. For enamels in which ‘pure’ oxides were detected as ‘impurities’, observation of the entire spectral window does not show any notable fluorescence, contrary to what is visible for the areas obtained with natural raw materials (cover used for the white areas of the heads, and light blue of the decor). Note the fine peaks (instrumental resolution) of the contribution of O_2_ and N_2_ gases trapped in bubbles at 1555 and 2330 cm^−1^, respectively. The high crystalline quality of the spinel observed for the black areas is consistent with a preliminary preparation based on chemicals; the moderate fluorescence is undoubtedly due to the contribution of natural organic matter allowing the installation of this decoration. The observation of the characteristic doublet of carbon (~1350–1585 cm^−1^) indicates an incomplete elimination of the organic matter during the firing.

The spectra obtained for this object are very representative of those obtained for late 19th century–mid-20th century enamels carefully prepared from chemicals. The pigments of the enamel on copper plate (Figure 11) are comparative with lead-arsenic apatite opacification and the use of hematite for a red coloration. Rather flat backgrounds are observed up to ~2500–3500 cm^−1^, free of narrow luminescence peaks.

With the exception of enamels colored by metallic nanoparticles, such as Au° and Cu° (e.g., pink to red colored areas, Figure 11), the spectra of enamels from the 19th century productions (see further for the 18th century) generally do not show significant fluorescence and in particular only represent exceptionally rare earth luminescence signatures, likely due to the rare earth impurities in the raw materials. White opacification was obtained either with cassiterite (Table 2, Figure 12) or lead arsenates (Table 2, Figure 11). If the pigments are very comparable at the first examination (minor but characteristic differences are present), the signatures of the silicate matrices differ according to the firing temperature. It is well established [18,19,31,32,33,34,35] and referenced herein that the variability of the polymerization network of a silicate determines the melting temperature and viscosity, etc., but also the Raman signature. In the case of a refractory silicate glass (e.g., porcelain glaze melting at 1400 °C), close to pure silica, which is completely polymerized (all SiO_4_ tetrahedra share their oxygen ions), the (symmetrical) SiO_4_ deformation mode is the most intense. On the contrary, in a very depolymerized glass, such as a glass rich in lead melting at a low temperature (~700 °C), the symmetrical SiO_4_ mode is very intense. Thus, the Raman spectra of low-temperature fired enamels show a peak around 950–1000 cm^−1^ and those fired at intermediate temperature around 1050 cm^−1^, while the glazes fired with the porcelain body at temperature over 1250 °C have a stretching component around 1150 cm^−1^.

### 5.4. 18th Century Pigments

As indicated in Table 2, the phases used as ancient pigment are corundum (hematite, red), pyrochlore (Naples yellows), apatite (lead arsenate white) and spinels (black). The minor differences concern the linewidth and limited shifts of the peak positions and components because these pigments consist of non-stoichiometric structures with partial substitutions of certain elements, such as Fe by Ti and Al for hematite, Fe by Mn, Cr and Ni for ferrites, Sn by Sb, Fe, Si and Zn in the Naples yellows, etc. Minority phases (wollastonites, calcium phosphates, sphene) characterize certain enamels and may be used as factory markers. Specifically, pigments used in the 17th and 18th centuries have received considerable attention [2,3,15,21,22,23,24,25,26,27,28,44,45,52,53,64,69,72,73,74,75,76,77,78,79,80,81,82,83,84]. However, the number of new pigments is limited. The most significant ones have the certain composition of Naples yellow pyrochlore (addition of Sb and Zn to the old Pb_2_Sn_2_O_6_ pigment) and arsenate apatite. In fact, these pigments are a 17th century innovation. As a result of arsenic being associated to cobalt in European mining ores, arsenate-based phases are considered as a signature of the use of European ores. However, the favorable effect of arsenic on the quality of cobalt blue rapidly led to voluntary addition of arsenic. In many cases, the blue is obtained using boron addition as a flux, as reported in the 18th century recipes [29,44]. Boron is difficult to detect using most analytical techniques. Due to the high covalent character of the BO3 entities, their Raman signature is well detected at ~1270–1290 cm^−1^, as shown in Figure 13. According to the use of raw materials and impure chemicals, a fluorescence background is generally observed over 1500 cm^−1^. Narrow luminescence peaks are exceptional and very characteristic of the raw materials used [43,61].

## 6. Conclusions

It is now well established that the identification of specific “Raman” signatures (external and internal fundamental modes of vibration) can contribute to the dating and authentication of artefacts by discussing the coherence of phases/elements identified by scholars and the use of three of the five-senses, such as sight, touch and sound. Elemental compositions recorded using X-ray fluorescence (mobile or fixed set-up) support Raman assignments and specific impurities can trace the origin of raw materials with particular geological orogenesis. [21,22,23,24,25,26,64] Certainly, when fragments are available, valuable quantitative elemental composition is obtained by SEM-EDS (e.g., [23,44,85]) or LA-ICP-MS (e.g., [86]). Consideration of the whole spectral signature, fluorescence background and luminescence signals offer additional arguments. The identification of characteristic phases regarding a specific period or recipe (association of Mn with Co, As with Co, etc.) can then be used as a milestone. 

The identification of productions using the pigments developed and then used significantly from certain time periods gives post quem dates as follows.

- Naples yellows with complex composition (addition of Sb, Zn, etc.) from (the end of) the 17th century.
- Opacification with arsenic from the end of the 17th century.
- The highlighting of pigments based on chromium oxide is typical of the 19th century (e.g., Victoria green and sphene pink).
- Pigments of various colors using zircon, cassiterite and rutile as a pigment matrix and CdS-CdSe are typical of the years after 1960.

In addition, non-Raman scattering signatures are obtained, especially in the spectral domain beyond where the fundamental modes are observed.

- The very intense fluorescence of a red to orange enamel is characteristic of coloration with nanoparticles, copper (Cu°) or gold (Au°) or the solid solution of CdS-CdSe, which was tested in the early 20th century as a colorant for glasses, although created at the end of the 19th century for paint pigments. In fact, it was only used at large scale in the first quarter of the 20th century for glass (stained glass windows) [41,42] and after 1950 for enamels [39].
- Strong ‘broadband’ fluorescence contributions are observed for some enamels prepared from natural raw materials while those obtained from purified reagents show fluorescence-free spectra. It is an index of ingredients prepared between ~1850 and 1960.
- Narrow luminescence peaks are frequent for enamels/glazes prepared in the second half of the 20th century, containing rare earths deliberately added or resulting from pollution by grinding agents.

It is significant to note that certain phases were only well detected with a blue laser, such as in the case of borates. Furthermore, the observation of the signatures of amorphous silicates is also optimized under blue excitation and becomes difficult using red excitation and a fortiori infrared excitation.

Another important limitation of Raman analysis is the difficulty of highlighting certain phases. This is the case of tridymite (SiO_2_) which is very difficult to detect, more difficult than mullite, even when significant quantities are observed by diffraction [69]. The second difficulty concerns the strongly colored phases formed of elements with multiple variances which are heated up under the laser beam and transform, resulting in oxidation and phase transition [51,66,70,71]. The measurement must be done under very low laser power, which is expensive in terms of measurement time. If the exploration in-depth up to several tens or even certain microns is possible when the glass is not very colored, this measurement is also long and therefore could rarely be done on site given the availability of the objects being limited to days when the museums are not open to the public.

## Figures and Tables

**Table 1:** Representative artifacts currently and previously studied.

Period	Origin	Artifact	Type	References	This Work
18th century	Vincennes (France)	Palette cup	porcelain	[21]	
	Sèvres (France)	Coffee cup	porcelain	[21]	
	Comte d’Artois factory(France)	Vase	porcelain	[22]	
	China	Dish	porcelain	[23]	X
	China	Dish	porcelain	[23]	X
	China	Tea pot	porcelain	[24]	
	China	Bottle	porcelain	[24]	
	Swiss	Watch	gold	[15]	
	France	Watch	gold	[15]	X
	China	Ewer	gold	[25,26]	
	France	Figure	glass	-	X
19th century	Sèvres (France)	Palette	porcelain	[2]	
	Sèvres (France)	Ewer	metal	[27]	
	Sèvres (France)	Palette	porcelain	[2]	
	Nevers (France)	Cup	Faience	-	X
	Satsuma (Japon)	Vase	porcelain	-	X
	China	Pendant	metal	-	X
	U.K. (Cobridge)	Tea cup saucer	faience	-	X
	Germany	Figure (peddler)	porcelain	-	X
20th century	Sèvres (France)	Vase	porcelain	[28]	
	Sèvres (France)	Palette	porcelain	[2]	
	China	Dish	porcelain	-	X
	Rosenthal studio line (Germany)	Coffee cup saucers (Cupola nr30)	porcelain	-	X
		Coffee cup saucers(Suomi Jahretasse 1999)	porcelain	-	X
		Coffee cup saucers(Salome nr17)	porcelain	-	X
		Coffee cup saucers(O. Alt nr7)	porcelain	-	X
	Japan	Sake cup (black)	porcelain	-	X
	Vietnam	Vase	porcelain	-	X
	China	Figure (Buddha))	porcelain	-	X

**Table 2:** Structure, formula, main characteristic Raman peak(s) or doublet wavenumber of crystalline pigments; period of use is given.

Color	Structural Type	Formula ^1^	Main Raman Peaks ^3^/cm^−1^	Period of Use/Century
Opacifier(white)	Cassiterite ^2^	SnO_2_	635–775	Roman
	Baddaleyite	ZrO_2_	180–190	20th
	Zirconia (stabilized)	ZrO_2_:Ca,Mg	265	20th
	Apatite	(Na,K,Ca)^1^Pb_4_(AsO_4_)_3_	815	<18th
	Rutile ^2^	TiO_2_	445–610	20th
	Zircon	ZrSiO_4_	1009	20th
	Whitlockite	Ca_3_(PO_4_)_2_	965	<17th
	Wollastonite	CaSiO_4_	635–970	18th
	Fluorite	CaF_2_	320	Medieval
		CaSb_2_O_6_	671	Roman
		CaSb_2_O_7_	482–633	Roman
Yellow	Pyrochlore	Pb_2_(Sb,Sn,Fe,Si)^1^_2_O_7-__δ_	130 to 140	Renaissance
		Pb(Sn,Fe,Si)^1^O_4_	130 to 140	Antiquity
	Zircon	(Zr,V)^1^SiO_4_	1009	>1960
	Zircon	(Zr,Pr)^1^SiO_4_	1009	>1960
	Baddeleyite	(Zr,V)O_2_	180–190	20th
	Rutile	(Ti,Ni,S)^1^O_2_	445–610	20th
	Cassiterite	(Sn,V)O_2_	635–775	20th
	Wurtzite	CdS	275	20th
		PbUO_4_	circa 830	19th
	Sphene	CaSnSiO_5_	580	20th
		ZnCrO_4_	840	>~1850
Blue	Hauyne, lazurite ^4^	Na_8_(Al_6_Si_6_O_24_)S_n_	542–1090	Antiquity
	Zeolite	Na_8_(Al_8_Si_8_O_32_)S_n_		19th
	Olivine	Co_2_SiO_4_	810–830	18th
	Phenacite	(Co,Zn)^1^_2_SiO_4_		19th
	Spinel	CoAl_2_O_4_	690	End of 18th
	Spinel	(Co,Zn)^1^Al_2_O_4_	690	19th
	Zircon	(Zr,V)^1^SiO_4_	1009	>1950
		BaMnO_4_	?	20th
Green	Yellow pigment in blue matrix	See above		18th
	Garnet	3CaO^.^Cr_2_O_3_^.^3SiO_2_	~750	data
	Olivine	Ni_2_SiO_4_	~850	20th
	Corundum	Cr_2_O_3_	~540	19th
	Spinel	Co(Cr,Ti)^1^_2_O_4_	700–800	19th
Red	Corundum ^2,4^	(Fe,Al,X)^1^_2_O_3_	200–300	Antiquity
	Wurtzite	CdSe	190	20th
	Wurtzite	Cd(S,Se)^1^	190–275	20th
	metal	Au°	-	17th
	metal	Cu°	-	Roman
Pink	Sphene	CaO^.^SnO_2_^.^SiO_2_:Cr	750–940	19th
	Corundum	(Al,Mn)^1^_2_O_3_	~420–750	20th
Blue	Spinel	Zn(Al,Cr)_2_O_4_	630–850	<18th
	Zircon	(Zr,Fe)^1^SiO_4_	1009	20th
Gray	Cassiterite ^2^	(Sn,Sb)^1^O_2_	635–775	20th
Brown	Spinel	Fe_2_TiO_4_	650–700	20th
	Spinel	(Zn,Ni,Fe)^1^FeO_4_	650–700	<18th
	Rutile	(Ti,Mn,Cr,S,Nb)^1^O_2_	450–600	20th
Black	Spinel	CuCr_2_O_4_	450–600	19th
	Spinel ^4^	(Co,Fe)(Fe,Cr)^1^_2_O_4_	450–600	<18th
	Spinel ^4^	(Fe,Mn)(Fe,Cr,Mn)^1^_2_O_4_	450–600	<18th
		CuO	300–350	<18th
	disordered	C	1350–1590	<18th

^1^ The relative content of elements given within brackets is not given; they vary from less than 0.01 to 0.5. ^2^ The structure of rutile and cassiterite are similar; hematite belongs to corundum structure. ^3^ Representative wavenumber of the stronger peaks; wavenumber can be shifted as a function of the laser excitation wavelength and precise non-stoichiometry/composition. ^4^ Synthetic or natural compound.

## Data Availability

Not applicable.
